# Global research landscape of HIV and Kaposi’s sarcoma: a visualized bibliometric analysis

**DOI:** 10.3389/fmicb.2025.1601245

**Published:** 2025-06-25

**Authors:** Shengwei Ma, Xuannan Chen, Yu Lai

**Affiliations:** ^1^Acupuncture and Tuina School, Chengdu University of Traditional Chinese Medicine, Chengdu, China; ^2^School of Basic Medicine, Chengdu University of Traditional Chinese Medicine, Chengdu, China

**Keywords:** HIV, Kaposi’s sarcoma, CiteSpace, bibliometric analysis, visualized analysis

## Abstract

**Background:**

Human immunodeficiency virus (HIV) infection remains a significant global health challenge, with Kaposi’s sarcoma (KS) being one of the most common AIDS-defining cancers. This study aimed to investigate the development of HIV and KS (HIV-KS) research through bibliometric analysis and to assess the current literature to advance the field.

**Methods:**

We gathered 1,568 publications on HIV-KS from the Web of Science Core Collection and analyzed them to visualize the data, identifying future directions and emerging trends by CiteSpace software. Betweenness centrality, count, and burst value were used as metrics.

**Results:**

The number of publications on HIV-KS fluctuated in the included literature. The most important contributions from countries and institutions were the United States and the National Institutes of Health (NIH) – USA, respectively. Denise Whitby was the most prolific author, while Yuan Chang had the highest cocitation count. The most commonly cited article is “Kaposi sarcoma (2019).” The hotspots in this field are “basic mechanism,” “Kaposi’s sarcoma-associated herpesvirus-related disease,” and “treatment measures.” The present research direction of HIV-KS is focused on exploring the emerging prevalence, diagnostic methods, and therapeutics.

**Conclusion:**

This study outlines the current landscape and emerging hotspots of HIV-KS research, offering insights into thematic evolution and collaboration patterns. By identifying key research priorities and structural gaps, the findings can inform future scientific directions, guide resource allocation, and support more context-sensitive strategies for the diagnosis, treatment, and prevention of HIV-KS.

## 1 Introduction

Human immunodeficiency virus (HIV) is one of the infectious pathogens that poses a severe threat to global health. The virus has a prolonged incubation period, exhibits high variability, and increases susceptibility to various illnesses, including various cancers. By the end of 2023, 39.9 million people were living with HIV (PLWH), and the virus had caused 42.3 million deaths worldwide (HIV Data and Statistics, n.d.). Although combination antiretroviral therapy (cART) has significantly improved patient survival, numerous comorbidities, particularly AIDS-defining cancers, continue to impact the health and mortality of PLWH seriously. Multiple regional studies have demonstrated that among various diseases, Kaposi’s sarcoma (KS) has the highest standardized incidence ratio (SIR) in PLWH, significantly exceeding that of other conditions (Galceran et al., 2007; Hernández-Ramírez et al., 2017; Lee et al., 2022). This finding suggests a strong link between KS and immunosuppression. PLWH experience a considerable drop in CD4+ T cells, leading to gradual impairment of immune function, thereby increasing the risk of KS.

In 1994, researchers at Columbia University identified a novel gamma-herpesvirus DNA sequence in KS lesions, subsequently termed Kaposi’s sarcoma-associated herpesvirus (KSHV), also known as human herpesvirus 8 (Mesri et al., 2010). Current epidemiological projections for 2022 indicate approximately 35,813 incident KS diagnoses and 16,169 related deaths worldwide, with 86.1% of deaths concentrated in Africa (Cancer Today, n.d.). Effective prevention and treatment remain significant challenges, especially in regions with high HIV prevalence. KS is also a clinical hallmark of acquired immunodeficiency syndrome (AIDS), indicating severe immune system compromise. The incidence of KS in PLWH, notably in HIV-infected men, is substantially higher than in people in general. KS originates in the lining of blood and lymphatic vessels and is associated with KSHV (Ceccarelli et al., 2019). Like other herpesviruses, KSHV undergoes a biphasic life cycle with latent and lytic replication phases (Carneiro et al., 2022; Soto et al., 2022). The primary transmission routes of KSHV are sexual and bloodborne, which may explain the high prevalence of KS among PLWH. Transmission through saliva and the possibility of iatrogenic transmission has also been reported. Recent studies have also shown that KSHV interacts with Epstein–Barr virus (EBV), contributing to the rising incidence of lymphomas among PLWH in developed countries, although the underlying mechanism remains to be elucidated (Han et al., 2024). To further investigate the relationship between HIV and KS (HIV-KS) and advance research in this discipline, a systematic evaluation and overview of existing studies is necessary. This is urgent to better explore the pathogenesis of KS, optimizing current treatment strategies, and developing new therapeutic targets and vaccines.

Bibliometrics is an interdisciplinary field in which analytical methods are utilized to examine scholarly outputs along with associated metadata like abstracts, keywords, and citations to clarify the connections between various academic publications (Broadus, 1987). Nowadays, bibliometrics has increasingly been adopted across various fields to assess and evaluate their respective progress and research frontiers. Professor Chaomei Chen developed CiteSpace, a scientometric knowledge domain visualization tool based on Java, which has become a significant resource for academic research (Chen, 2004). CiteSpace relies on co-citation analysis theory and betweenness centrality, among other methodologies, integrating bibliometrics, visualization techniques, and data mining algorithms to perform visualization tasks (Chen, 2006). In recent years, scholars have used CiteSpace to summarize key areas of HIV research, including HIV-MTB co-infection, pre-exposure prophylaxis, and immune activation, which have significantly advanced the field of HIV research (Chen and Lai, 2023; Gong and Lai, 2023; M et al., 2023). In this context, this study seeks to utilize CiteSpace to investigate the following research question: What are the global research trends, collaboration patterns, and thematic developments in HIV-KS research over the past decade? To address this, we aim to fill a critical knowledge gap by systematically mapping and visualizing the global research landscape.

## 2 Methods

### 2.1 Data collection and processing

Data were gathered using the advanced search feature of the Web of Science Core Collection (WOSCC) with the following search terms: [TS = (HIV) OR TS = (“Human Immunodeficiency Viru*”) OR TS = (HTLV-III) OR TS = (“AIDS Viru*”) OR TS = (LAV-HTLV-III) OR TS = (“Lymphadenopathy-Associated Viru*”) OR TS = (“AIDS Viru*”)] AND [TS = (Sarcoma, Kaposi) OR TS = (Kaposi Sarcoma) OR TS = (Kaposi’s Sarcoma) OR TS = (Kaposis Sarcoma) OR TS = (Sarcoma, Kaposi’s) OR TS = (Multiple Idiopathic Pigmented Hemangiosarcoma)]. As WOS is the only data source that fully supports the complete range of CiteSpace’s analytical features (Gong and Lai, 2023), and in the search rules of WOSCC, “TS” denotes the topic within the search formula. Literature types such as conference proceedings, newspapers, and books were excluded. Only articles and review articles published in English were included. The selected publications spanned the period from January 1, 2011, to December 31, 2024.

**Figure 1:**
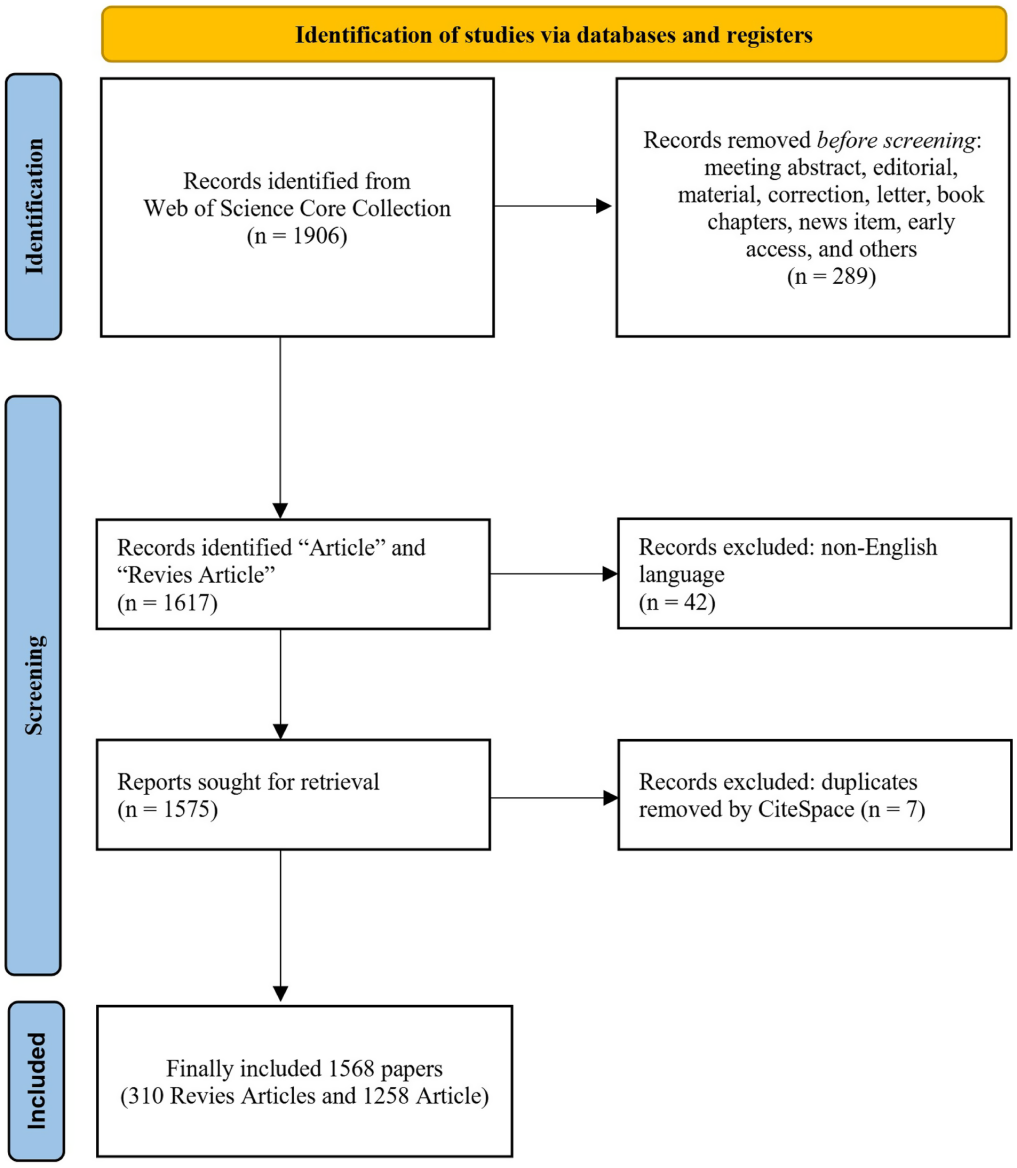
Retrieval process. This figure presents a flowchart illustrating the literature retrieval method for our investigation.

This study utilized CiteSpace software version 6.4.R1, with data collection completed on January 23, 2025. The retrieval process is illustrated in Figure 1. All three investigators independently evaluated the data obtained from the WOSCC and exported the literature in the “full records and cited references” format. Data from records 1–500 were saved in a file named “download_01.txt,” records 501–1,000 in “download_02,” and so on, until all relevant data were exported. A total of 338 irrelevant or duplicate articles were removed through manual screening and CiteSpace’s “Remove duplicates” function. Ultimately, 1,568 articles were included in the final dataset.

### 2.2 Bibliometrics and visualization analysis

After importing all the available data into CiteSpace, the time span was set, and “Years Per Slice” was selected as 1 year. The g-index is a metric for controlling network node density and filtering influential literature. Within each time slice, the node selection criterion follows Equation 1:


(1) $$g^{2}\leq k\sum_{i\leq g}c_{i},k\in Z^{+}$$

$\sum c_{i}$ represents the cumulative citation count of publications, reflecting their aggregated scholarly impact. The parameter *k* functions as a scaling factor to balance analytical comprehensiveness and focus. Increasing the *k*-value expands the network by relaxing citation thresholds while decreasing it prioritizes high-impact nodes. In our analysis, the scaling parameter *k* was maintained at its default value of 25, with all other configurations preserved, including the log-likelihood ratio (LLR) method for cluster labeling. This standardized setup ensures reproducibility while mitigating subjective bias in network construction. Modularity (*Q*) serves as a metric to evaluate the quality of clusters, calculated as shown in Equation 2:


(2) $$Q=\frac{1}{2m}\sum_{\text{ij}}(a_{\text{ij}}-p_{\text{ij}})\sigma (C_{i},C_{j})$$

*m* represents the total number of edges in the network, $a_{\text{ij}}$ represents the actual number of connections between nodes *i* and *j*, $p_{\text{ij}}$ is the expected number of it, and $\sigma (C_{i},C_{j})$ is an indicator function that equals 1 if nodes *i* and *j* belong to the same cluster, or 0 otherwise. Its values range from 0 to 1, with higher values indicating better clustering performance.

In CiteSpace, information such as countries, institutions, authors, and keywords are represented as nodes, with their size corresponding to frequency and color signifying the year of occurrence. Lines between nodes indicate the strength of collaboration or co-occurrence. Nodes with bright borders are typically considered high centrality (centrality ≥ 0.1) (Chen et al., 2024). In graph theory, centrality quantifies a node’s structural importance within interconnected systems, defined as Equation 3:


(3) $$g(v)=\sum_{s\neq v\neq t}\frac{\sigma_{\text{st}}(v)}{\sigma_{\text{st}}}$$

The centrality metric g(v) quantifies node *v*’s structural significance, where $\sigma_{\text{st}}(v)$ reflects the frequency of *v*’s involvement in minimal-length pathways linking nodes *s* and *t*, while $\sigma_{\text{st}}$ enumerates all such optimal routes between the pair. Burst-analysis techniques significantly recognize abrupt surges in research interest within a specific domain (Lai et al., 2024). In each figure, the Look Back Years (LBY) parameter sets the maximum backtracking period, ensuring that only past N-year references are included. Additionally, the Edge Count (E) reflects the total number of connections in the network, with larger E values indicating a higher density of connections between nodes. Density reflects the compactness of connections among nodes in a network, where higher values suggest a more tightly interconnected structure. Larger nodes represent more publications or citations. To methodically tackle the research inquiry, our analysis connects three core dimensions with specific bibliometric indicators, as presented in Table 1.

**Table 1:** Research objectives and corresponding bibliometric indicators.

Research question	Analysis dimension	Key metric
Identification of research hubs	Country/Institution networks	Betweenness centrality
Impact of major contributors	Author/References/Journal cocitation	Cocitation Count/Frequency Betweenness centrality Cluster
Evolution of research themes	Keywords	Co-occurrence frequency Burst strength, Cluster

## 3 Results

### 3.1 Analysis of annual production of publications

**Figure 2:**
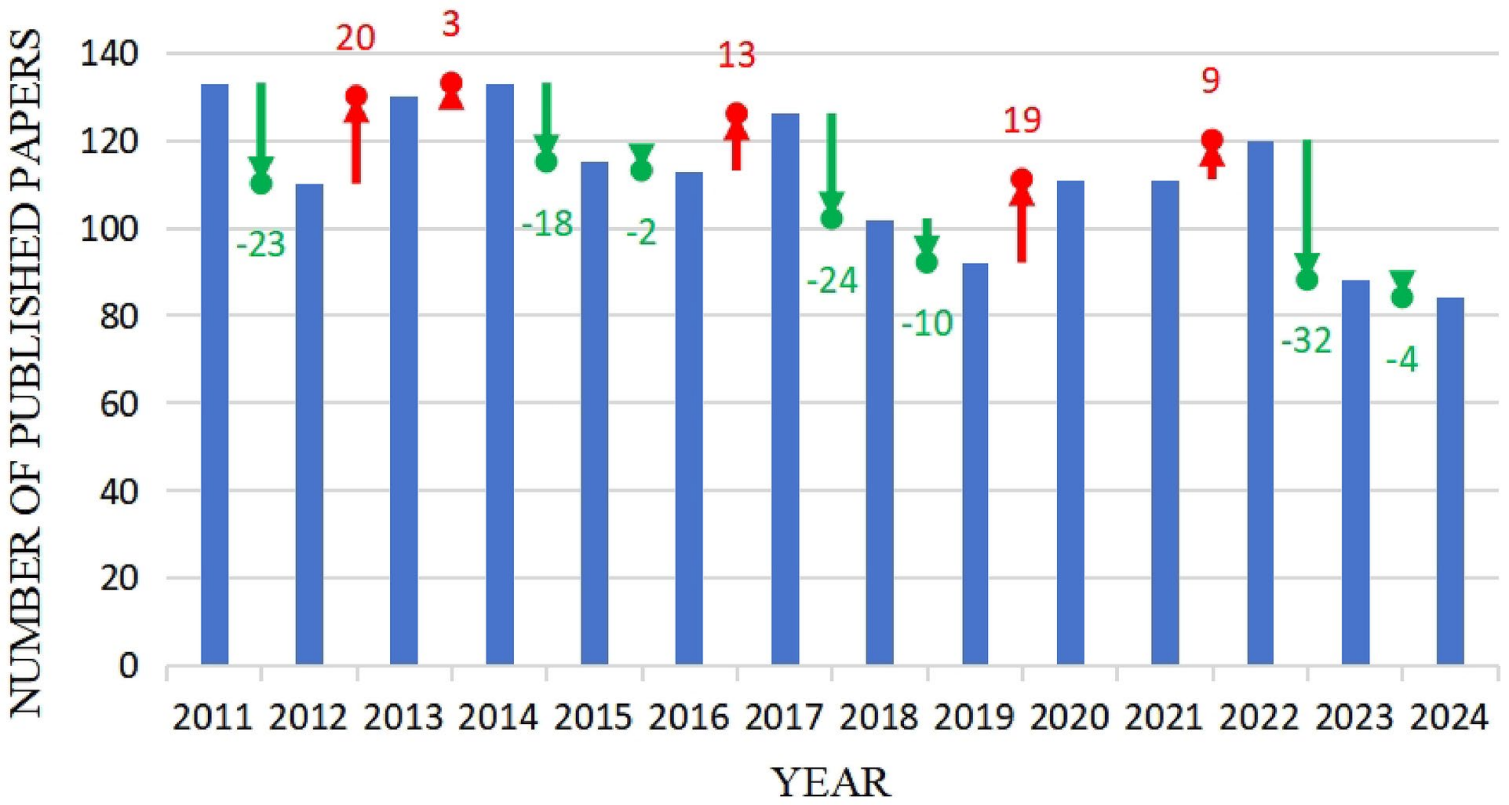
Annual chart of publications. This figure shows a composite image of the bar chart of the annual publishing volume and the scatter plot of the increasing and decreasing trends.

To visualize the distribution of publications in HIV-KS research, we plot the year of publication on the X-axis and the number of publications on the Y-axis. This approach allows us to observe the trends in the development of research output over time, revealing observations about historical patterns and enabling predictions for future growth in the subject, as clarified in Figure 2. The graph reveals an overall fluctuating trend in the number of publications. The earliest article included in this analysis, published by Senba et al., proposed that the rising incidence of KS might be linked to HIV infection (Senba et al., 2011). This fluctuation may result from the widespread use of ART, leading to reduced clinical research demands. Additionally, shifts in scientific priorities during the COVID-19 pandemic and a lack of breakthroughs in basic mechanisms have further dampened motivation for subsequent studies. Based on the trend of annual publication volume, efforts should be made to innovate or shift the research direction within this domain to attract greater scholarly attention and funding.

### 3.2 Analysis of countries and institutions

Table 2 presents the publication volume of the top 10 countries and institutions. These countries and institutions play important roles in the research, with the United States leading (756, 48.21%), followed by Italy (152, 9.69%) and the United Kingdom (136, 8.67%). The top three institutions in terms of publication volume are the National Institutes of Health (NIH) – USA (427, 27.23%), the University of California System (289, 18.43%), and Assistance Publique Hopitaux Paris (APHP) (149, 9.5%). More than half of these publications were from the United States and Italy (57.91%), published in the NIH – USA, the University of California System, and the University of California System (54.9%).

**Table 2:** Top 10 countries and institutions with published articles.

Rank	Count	Country	Count	Institution
1	756	USA	427	National Institutes of Health (NIH) – USA
2	152	ITALY	289	University of California System
3	136	ENGLAND	149	Assistance Publique Hopitaux Paris (APHP)
4	124	PEOPLES R CHINA	140	University of North Carolina
5	118	FRANCE	119	Harvard University
6	108	SOUTH AFRICA	118	University of Washington
7	79	UGANDA	70	University of London
8	74	BRAZIL	66	Institut National de la Sante et de la Recherche Medicale (Inserm)
9	66	GERMANY	44	Johns Hopkins University
10	58	SWITZERLAND	43	Universite Paris Cite

**Figure 3:**
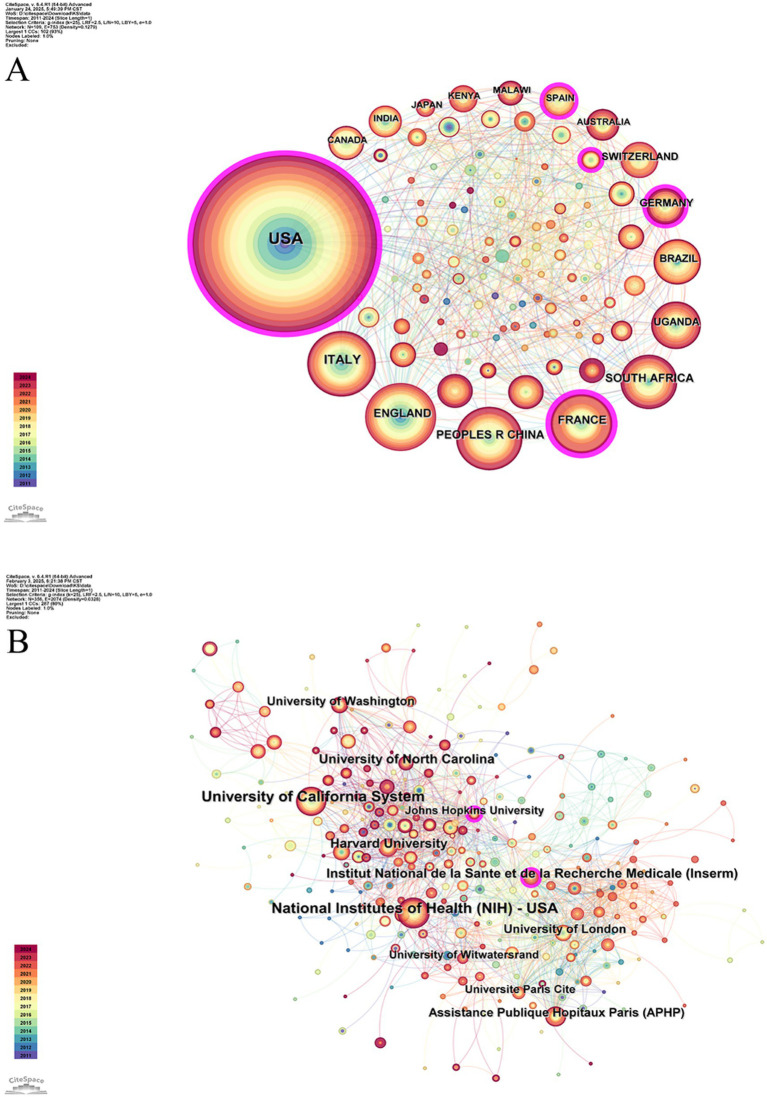
**(A)** Map of countries. **(B)** Map of institutions. The color gradient in the lower left corner indicates publication years, ascending chronologically from bottom to top. The magnitude of the circle represents the published volume by countries or institutes. The lines connecting the circles signify international collaboration among countries or institutions.

The construction of two network diagrams, one for countries and one for institutions, was facilitated to analyze collaboration patterns. Figure 3A shows the cooperation network among countries, with node size representing the number of articles published in each country. The lines between the nodes indicate inter-country collaboration. The network consists of 109 nodes and 753 connections, focusing on the strong cooperation among countries, particularly the United States, and its central role in the global collaborative network. Additionally, the purple borders around the nodes represent high centrality, identifying key countries like the United States, France, and Germany, which serve as vital hubs in the research network. These countries have a strong international presence in the study, facilitating academic exchanges and the dissemination of knowledge. Figure 3B presents the inter-institutional collaboration network, containing 356 nodes and 2,074 connections. This figure shows the tight collaboration between institutions, particularly those represented by NIH – USA. Institutions such as Johns Hopkins University, highlighted with purple borders, further emphasize their close interconnections within the research community. These findings provide valuable knowledge of the worldwide scope of research partnerships in this field and convey important references for future investigations and collaboration.

The United States accounted for the highest publication volume, which may be attributed to its historical research capacity and institutional infrastructure. The minimal institutional influence of sub-Saharan Africa in HIV-KS research reflects considerable limitations in finance and research capabilities. This has resulted in limited context-specific evidence to guide clinical care, persistent dependency on external expertise, and missed chances to customize therapies to local requirements. Addressing these gaps requires continuous funding and equitable collaborations that support local research systems.

### 3.3 Analysis of authors and cocited authors

The information in Table 3 offers an overview of the top 10 authors and cocited authors, ranked by either publication volume or cocitation count. Collectively, these top 10 authors have published a total of 289 articles (18.43%). Among them, Denise Whitby stands out as the most prolific author, with 52 published articles (3.31%). Regarding the cocitation count, Yuan Chang ranks first, with 386 articles citing multiple works by Yuan Chang, underscoring the significant influence and widespread recognition of the author within the field. Yuan Chang’s most recent contribution to this field used representational difference analysis to isolate KS agent DNA from an AIDS-KS tumor, identifying KSHV as the virus (Moore and Chang, 2014). The virus was localized to AIDS-KS tumors and absent from most non-KS tissues, whether or not the individuals were infected with HIV, providing significant proof for the etiology of AIDS-KS.

**Table 3:** Top 10 authors and cocited authors.

Rank	Count	Centrality	Authors	Count	Centrality	Cocited authors
1	52	0.07	Denise Whitby	386	0.01	Yuan Chang
2	37	0.05	Robert Yarchoan	315	0.03	Ethel Cesarman
3	32	0.03	Thomas S. Uldrick	284	0.01	Eric A Engels
4	28	0.05	Charles Wood	243	0.03	Thomas S Uldrick
5	27	0.15	Dirk P. Dittmer	241	0.05	Mark Bower
6	26	0.15	Eric A. Engels	223	0.02	Susan E Krown
7	24	0	Shou-Jiang Gao	176	0.03	Meredith S Shiels
8	22	0.02	Wendell Miley	165	0.03	A E Grulich
9	21	0.01	Zhiqiang Qin	163	0.02	J Soulier
10	20	0.01	Lu Dai	155	0.04	Antonino Carbone

**Figure 4:**
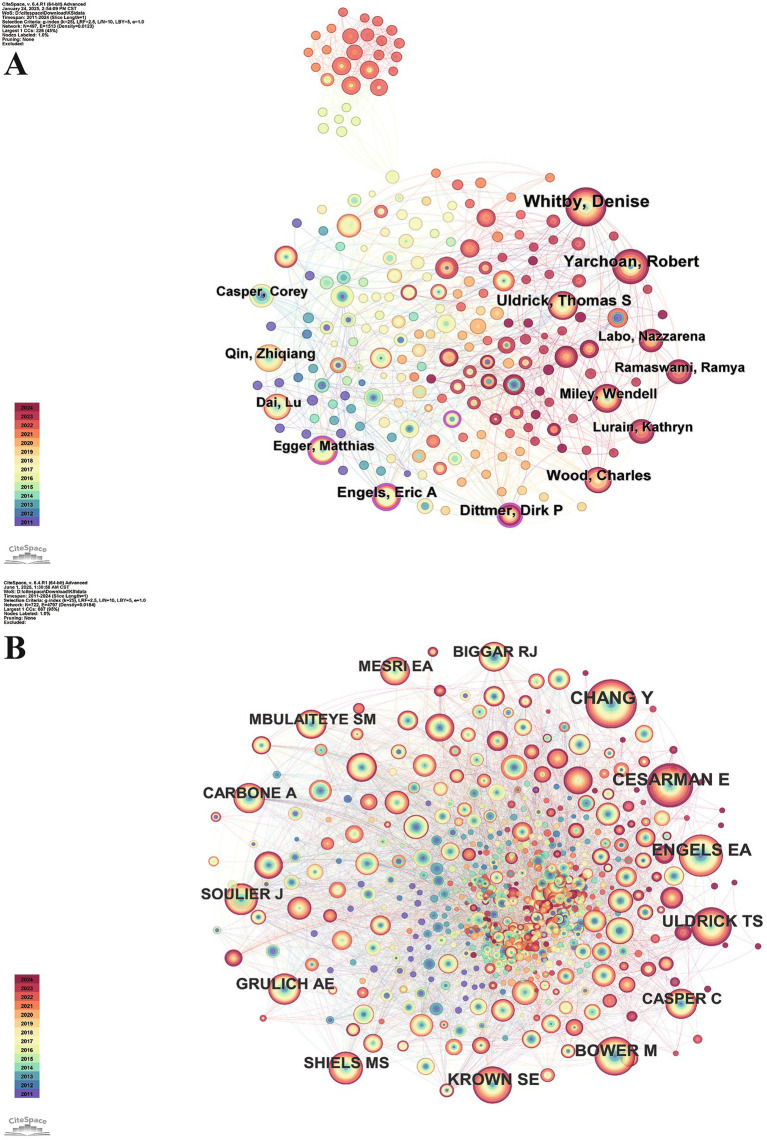
**(A)** Map of coauthors. **(B)** Map of cocited authors. The coauthors network illustrates active collaboration patterns among researchers in the field. Each node represents an author, with node size proportional to publication output and link thickness indicating the strength of collaboration. In the cocited network, nodes represent frequently cited authors, and their proximity reflects the degree of intellectual relatedness.

Figure 4A displays a coauthor network, where 497 nodes represent individual authors and 1,513 connecting lines indicate collaborations between them. Authors such as Dirk P. Dittmer (centrality = 0.15), and Eric A. Engels (centrality = 0.15), among others, have nodes with purple borders, indicating their high centrality and close collaboration with other authors in the field. We suggest that “peripheral authors”—those with relatively few publications but strong citation impact—may serve as early predictors of emerging frontiers. Tracking these signals could enable a more sensitive forecast of the next generation of HIV-KS research. We advise that future bibliometric techniques integrate a “peripheral burst index” to alert authors who demonstrate abrupt, intense co-citation activity, even if their overall publishing volume remains small.

Figure 4B presents the cocited author network, featuring 722 nodes and 4,797 connections. This visualization reveals the distribution of highly cited authors and helps identify key contributors within the HIV-KS research field. The absence of purple-bordered nodes indicates limited bridging authors. Betweenness centrality reflects how often an author lies on the shortest paths connecting others. Our CiteSpace analysis of HIV-KS coauthorship networks reveals high modularity (*Q* = 0.5668 > 0.3), indicating that most researchers collaborate within closely bounded subgroups rather than across them (Glynatsi and Knight, 2021). As a result, some highly productive authors remain on the network’s periphery and primarily drive advances within subfields without bridging distinct research clusters. This fragmentation highlights structural holes within the HIV-KS field. To address fragmentation in the HIV-KS research landscape, the field should adopt structural integration strategies that build lasting connections across research clusters. Funding agencies can promote this by supporting interdisciplinary projects, for example, pairing basic scientists studying KSHV latency with clinicians researching antiretroviral therapy outcomes. Investments in shared data infrastructure and meta-analyses can help unify disconnected literature and generate common conceptual models. Additionally, monitoring whether new studies involve authors from previously unlinked groups can help sustain integration. Closing these structural gaps will enhance the relevance of HIV-KS research and accelerate the uptake of new insights into the global knowledge system.

### 3.4 Analysis of cocited references

**Figure 5:**
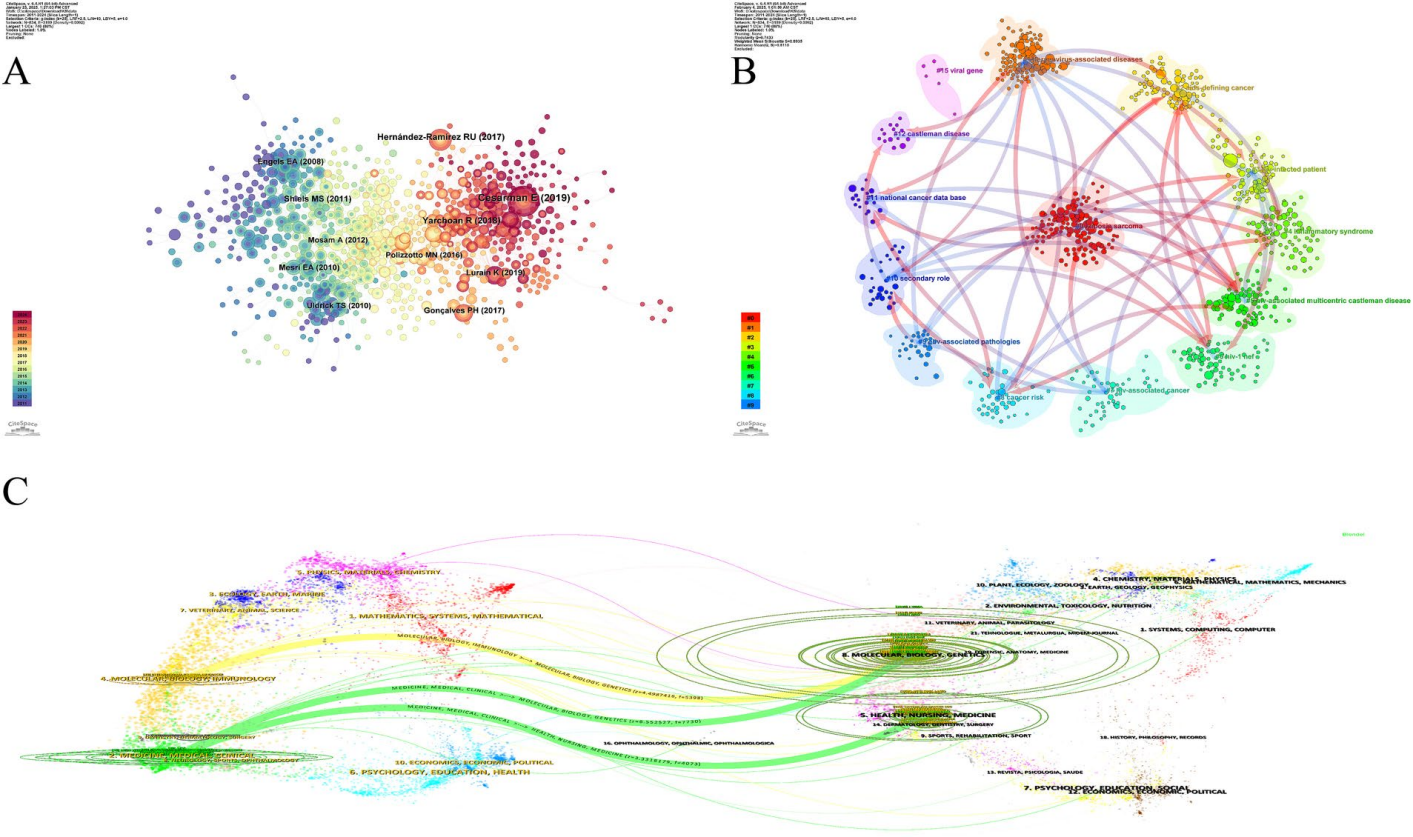
**(A)** Map of cocited references. **(B)** Cluster dependency map of the reference. **(C)** Overlay map of journals. In panel **(A)** each node represents one literature. There are 834 nodes and 3,199 lines in panel **(B)**. The node size is positively associated with the citation frequency of the literature. The line denotes the direction of dependence between clusters, and the thickness reflects the degree of the relationship between clusters. The cluster labels represent the number of each cluster and the core subject words. Panel **(C)** reflects the citation relationship across each research field.

Figure 5A shows a cocitation network with 834 nodes and 3,199 connecting lines. The cocitation relationship is established when two references are cited together in the same article. The top 10 articles are listed in Table 4. Compared to citation frequency, cocitation frequency more accurately reflects the value of references. References with high cocitation frequencies often serve as a solid foundation for long-term exploration within the field. In the graph, the differently colored nodes represent the years in which the cited literature was published, with nodes ranging from purple to dark red, indicating the progression from earlier to more recent publications. The two most cocited papers were comprehensive reviews published by Cesarman et al. (2019) and Yarchoan and Uldrick (2018). Robert Yarchoan and Thomas S. Uldrick highlight that HIV-induced immunosuppression weakens host control over KSHV, promoting KS development. Although ART significantly reduces KS incidence, individuals with restored CD4+ T-cell counts still face elevated risk, suggesting that immune reconstitution alone is not fully protective (Yarchoan and Uldrick, 2018). From a virological perspective, Cesarman et al. emphasize the role of KSHV latency genes, such as LANA, vFLIP, and viral microRNAs—in maintaining latency and promoting tumor cell survival and angiogenesis. They propose a “paracrine oncogenesis” model, in which cytokines like vIL-6 and VEGF, secreted by a small number of lytically infected cells, can drive transformation in neighboring cells (Cesarman et al., 2019). Collectively, these insights challenge the conventional view of KS as a clonal cancer, instead portraying it as a multicentric, virus-driven tumor shaped by the host immune context. The fact that ART alone can induce KS regression underscores its nature as an immune-reversible lesion. While ART remains the cornerstone of treatment, some patients still progress to advanced KS or develop IRIS-related worsening. Cesarman et al. also suggest that antiviral drugs like ganciclovir may offer preventive benefits, though therapeutic efficacy remains inconsistent. These findings suggest that KS is fundamentally distinct from typical mutation-driven cancers, raising important questions about whether its treatment priorities should shift from cytotoxic strategies toward immune restoration and viral suppression.

**Table 4:** Top 10 cocited references.

Rank	Count	Cocited reference	First author (year)
1	101	Kaposi sarcoma	Cesarman et al. (2019)
2	53	HIV-Associated Cancers and Related Diseases	Yarchoan and Uldrick (2018)
3	51	Cancer risk in HIV-infected people in the USA from 1996 to 2012: a population-based, registry-linkage study	Hernández-Ramírez et al. (2017)
4	46	Cancer burden in the HIV-infected population in the United States	Shiels et al. (2011)
5	38	Kaposi’s sarcoma and its associated herpesvirus	Mesri et al. (2010)
6	36	Cancer risk in people infected with human immunodeficiency virus in the United States	Engels et al. (2008)
7	36	Viral, immunologic, and clinical features of primary effusion lymphoma	Lurain et al. (2019)
8	34	HIV-associated Kaposi sarcoma and related diseases	Goncalves et al. (2017)
9	31	A randomized controlled trial of highly active antiretroviral therapy versus highly active antiretroviral therapy and chemotherapy in therapy-naive patients with HIV-associated Kaposi sarcoma in South Africa	Mosam et al. (2012)
10	31	Clinical Features and Outcomes of Patients With Symptomatic Kaposi Sarcoma Herpesvirus (KSHV)-associated Inflammation: Prospective Characterization of KSHV Inflammatory Cytokine Syndrome (KICS)	Polizzotto et al. (2016)
11	31	An interleukin-6-related systemic inflammatory syndrome in patients co-infected with Kaposi sarcoma-associated herpesvirus and HIV but without Multicentric Castleman disease	Uldrick et al. (2010)

Hernández-Ramírez et al. (2017) published the third most cocited paper. This study analyzed cancer risk among 448,258 PLWH across nine U.S. regions from 1996 to 2012, applying SIR and Poisson regression models. The study found that the overall cancer risk was 69% higher than the general population, with KS risk particularly elevated (SIR = 498) (Hernández-Ramírez et al., 2017). These publications deliver essential literature support for the study of HIV-KS, summarizing research advancements and emphasizing the strong association between them, which has caught the attention of scholars.

Dependency among clusters refers to specific clusters constructed based on other clusters, as shown in Figure 5B. For example, cluster #1 (polymer micelles) references several other clusters, including #0 (kaposis sarcoma), #2 (aids-defining cancer), #3 (hiv-infected patients), #5 (hiv-associated multicentric castleman disease), #6 clustering (hiv-1 nef), #8 clustering (cancer risk), #11 (national cancer data base), and #12 (castleman disease) in the literature, suggesting that clusters #0, #2, #3, #5, #6, #8, #11, and #12 are the knowledge base for cluster #1. This interconnectedness highlights how knowledge from various clusters contributes to the study of HIV-KS across different areas. Cluster #15 (viral genes) shows the weakest linkages with other clusters, possibly reflecting disciplinary fragmentation. This may be due to different aims between basic and clinical research, limited cross-field contact, high technical barriers, the cost of genetic research equipment, and the lack of effective mechanisms for sample and data sharing. One potential solution is to fund a small number of pilot projects that use viral genes as a point of entry and directly assess their clinical or public health relevance. For example, measuring the KSHV LANA gene with CD4/CD8 ratios or chemotherapy response could assist uncover early prognostic signals.

### 3.5 Analysis of journals: overlay map

Figure 5C was generated by CiteSpace’s overlay map tool. On the left side, the graph shows the categories of citing journals, while the right side shows the categories of cited journals, with curves connecting them to indicate the citation relationships. The ellipse curves reflect the ratio of authors to publications: the longer the horizontal axis, the more authors involved, and the longer the vertical axis, the more papers produced by the journal. On the citing journals side, the graph predominantly displays journals in the fields of molecular, biology, immunology, medicine, clinical, mathematics, and others. Correspondingly, the cited journals primarily originate from journals focused on molecular, biology, genetics, health, nursing, medicine, dermatology, dentistry, and surgery.

We believe that interdisciplinary spillover from domains like dermatology and dentistry can drive innovation in HIV-KS research. Specifically, dermatologic improvements in skin imaging, photodynamic treatment, and topical medication administration may produce novel ways for identifying and treating cutaneous Kaposi’s lesions. Similarly, dental research on oral mucosal diagnostics, saliva-based biomarkers, and minimally invasive lesion collection could give novel techniques for early identification of oral KS. By finding and funding “hub” papers that bridge virology with dermatology or dentistry, scientists can stimulate focused collaborations that transfer this related knowledge into improved HIV-KS outcomes.

### 3.6 Analysis of keywords

#### 3.6.1 Analysis of keyword co-occurrence

**Figure 6:**
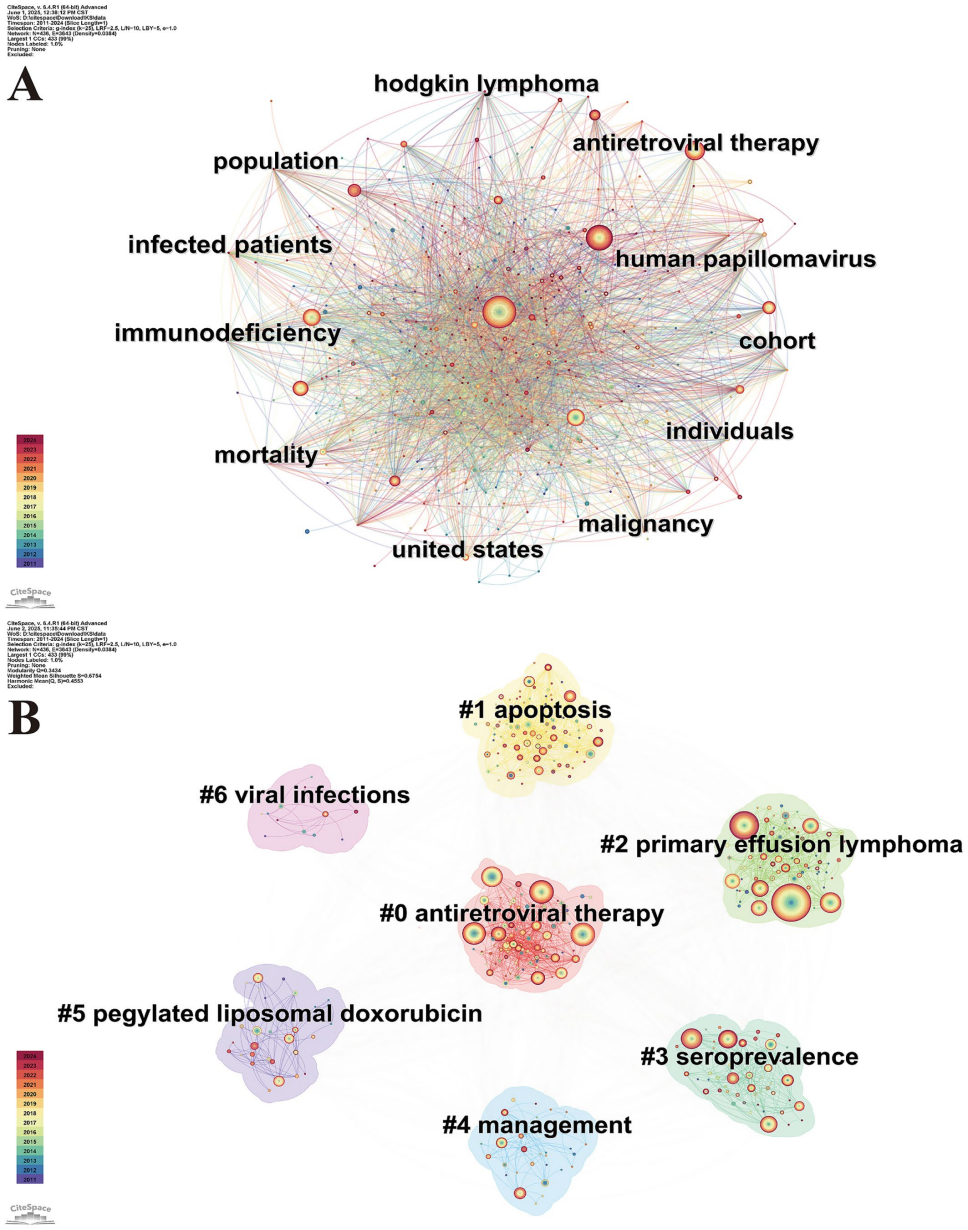
**(A)** Map of keyword co-occurrence. **(B)** Map of keyword clusters. Map of keyword co-occurrence illustrating the most frequently used terms and their interrelationships in the HIV-KS literature. The clustered keyword map displays automatically labeled groups based on thematic similarity, with each cluster representing a major research theme.

Keyword analysis helps to point out the core topics and emerging trends within the research field

offering a simplified summary of central research areas. A keyword co-occurrence map visualizes the relationships between keywords based on their frequency of co-occurrence. Figure 6A Displays the map, which includes 436 nodes and 3,643 connecting lines. The color gradient of the connecting lines from purple to red indicates the temporal proximity of the keywords, with red lines representing more recent co-occurrences. Prominent keywords in the map include “antiretroviral therapy,” “mortality,” “immunodeficiency,” and others. For example, in “immunodeficiency,” HIV in AIDS-KS directly induces immunodeficiency, while the KSHV-encoded products of the *K3* and *K5* genes inhibit antigen presentation through major histocompatibility complex, preventing the immune system from detecting KSHV-infected cells. Together, these factors increase the risk of disease at the immune level (Cesarman et al., 2019). All these terms reflect the central research topics and hot spots in the field. The HIV-KS literature not only tackles KS itself but situates it within a broader framework of HIV-related malignancies, coinfections, and epidemiologic research. Their prevalence in co-occurrence analysis suggests that future research should focus on the oncogenic role of viruses under immunosuppression, clinical cohorts, and population-level trends. This pattern coincides with broader directions in contemporary HIV research.

#### 3.6.2 Analysis of keyword clusters

Keyword clustering analysis is essential for identifying the structure and dynamics of the research field, as well as pinpointing emerging research frontiers. To perform this analysis, we utilized the LLR ranking algorithm in CiteSpace’s all-in-one function based on the keyword co-occurrence map. Figure 6B Presents seven clusters: #0 (antiretroviral therapy), #1 (apoptosis), #2 (primary effusion lymphoma), #3 (seroprevalence), #4 (management), #5 (pegylated liposomal doxorubicin) and #6 (viral infections). The evaluation of the clustering effectiveness relies on two key metrics: The average contour value (*S*) and the cluster modularity value (*Q*). *S* greater than 0.5 indicates reasonable clustering, with values exceeding 0.7 indicating high reliability. *Q* is crucial for assessing the rationality of the cluster structure. When *Q* is greater than 0.3, the community structure is considered significant. The larger the *Q* value, the more internal links within the clusters, indicating better clustering performance. Based on the keyword clustering results generated from the HIV-KS topics, it can be observed that our clustering is effective (*Q* = 0.3434, *S* = 0.6754). Thus, the seven knowledge clusters effectively reflect the distribution of research topics in the examined literature.

#### 3.6.3 Evolution of research focus

**Figure 7:**
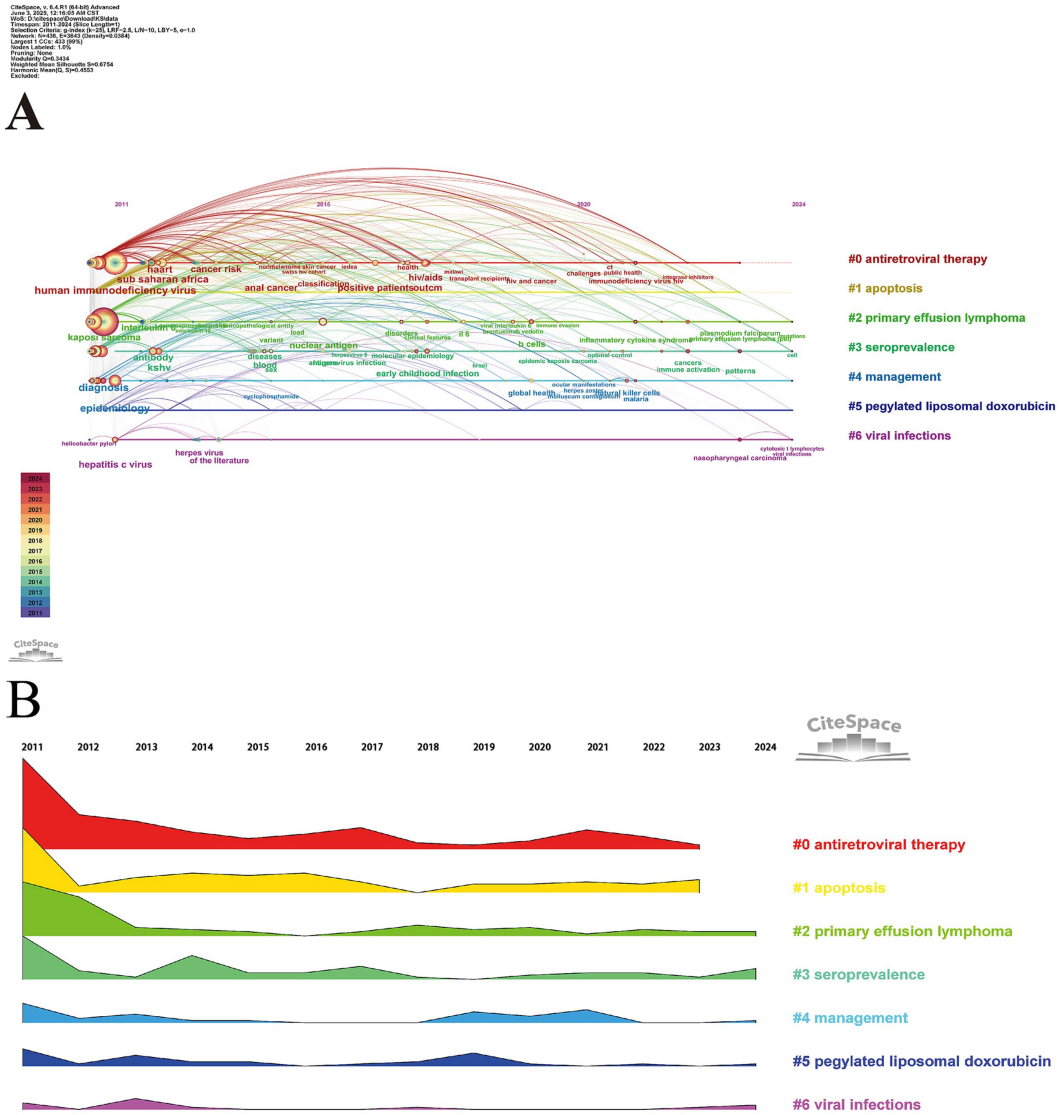
**(A)** Timeline view of the keyword co-occurrence map. **(B)** Landscape of clusters. Panel **(A)** demonstrates the composition of different keywords within each cluster across various periods. From panel **(B)**, we can intuitively understand the changing trend of the subject degree of each cluster during different times.

Figure 7 presents the results of the timeline and landscape view analyses, respectively, illustrating the evolution of research hotspots over time. Based on the data from these figures, research on HIV-KS has shown modest changes in recent years. Cluster #0 (antiretroviral therapy) and #1 (apoptosis) have remained prominent, although both have matured over the past 2 years. The reason for the perceived maturity in this field may lie in improved disease control and shifting research priorities. As treatment options have matured, the disease burden has decreased. Simultaneously, funding efforts have shifted toward vaccine development and other emerging health threats. To address this gap, renewed funding initiatives targeting HIV-KS biology and breakthroughs in the mechanistics are needed.

**Figure 8:**
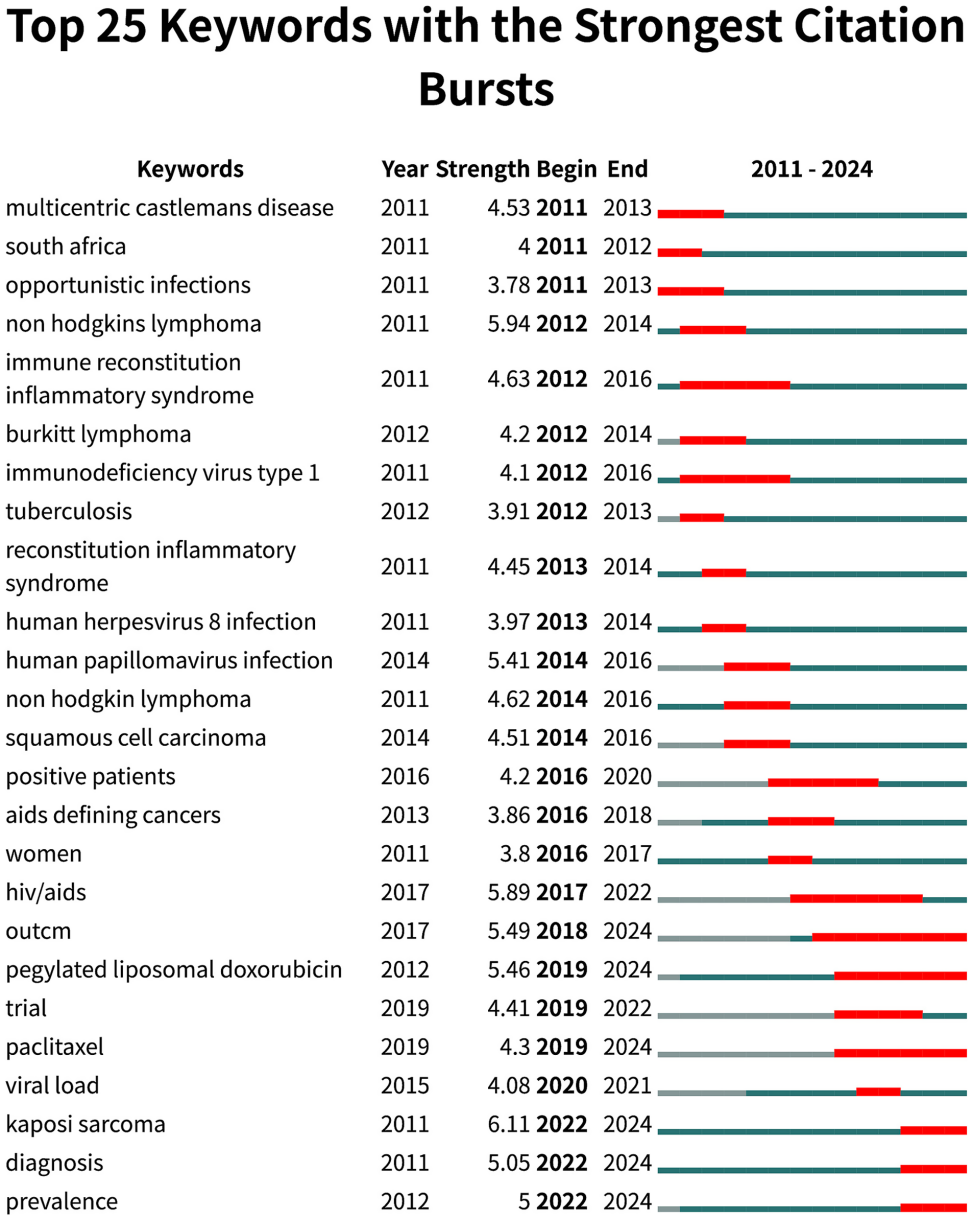
Top 25 keywords with the strongest citation bursts. This figure provides the first appearance time of each keyword, the time when the citation burst ended, and their relative intensity. The hot keywords that continue to the present can also be identified through the end time and the red line segment.

“Keywords with citation bursts” refers to a notable increase in the frequency of keyword usage during a specific period. By detecting and analyzing these burst keywords, we can identify the research frontiers of a given period and track their evolution over time, which helps predict future research trends and directions. In visual analysis, the blue line typically represents a particular moment, while the red line marks the period of keyword bursts. The top 25 HIV-KS-related terms identified through burst detection methods are shown in Figure 8. Some of these results align with the clustering outcomes previously discussed. Based on the initial appearance of keywords, the research includes terms such as “pegylated liposomal doxorubicin,” “paclitaxel,” “diagnosis,” and “prevalence” continue to exhibit strong citation bursts, while the continuous attention they attract is also consistent with the rising trends observed in the landscape view clusters #3 (seroprevalence) and #5 (pegylated liposomal doxorubicin), indicating potential future research directions.

These temporal shifts in keyword prominence closely reflect critical developments in the clinical management, research priorities, and epidemiological patterns of HIV-KS over the past decade. For instance, the increased frequency of terms such as “antiretroviral therapy” and “CD4/CD8 ratio” corresponds with the global expansion of cART. This advancement transformed HIV from a fatal illness into a manageable chronic condition, subsequently enabling researchers to shift their focus toward long-term complications such as KS. This shift is evident in the rising use of keywords like “mortality,” “management,” and “IRIS.” The emergence of “pegylated liposomal doxorubicin” and “paclitaxel” as burst keywords corresponds with their role as therapeutic agents in the treatment of KS. More recently, the growing prevalence of keywords such as “seroprevalence,” and “diagnosis,” underscores attention to early detection and surveillance in high-burden regions. These evolving thematic trends not only reflect shifts in scientific focus but are also shaped by clinical innovations, epidemiological dynamics, and global health policy initiatives.

## 4 Discussion

### 4.1 General information

#### 4.1.1 Overview of research results

In this study, a bibliometric analysis was conducted on 1,568 articles related to HIV-KS retrieved from the WOSCC between 2011 and 2024. The overall publication trend demonstrates a fluctuating trend, potentially due to continuous advancements in the field and the availability of well-established treatment strategies. This trend highlights the need for innovative research discoveries. Although North America and Europe dominate in institutional production, their centrality is low and interconnectedness is limited, suggesting that the greater potential for global collaboration has yet to be fully realized. The 10 most prolific authors contributed 289 papers; however, only two had centrality scores above 0.1. The coauthor network analysis indicates the presence of translational “choke points,” where only a limited number of persons act as intermediaries between basic scientific research and clinical practice. In summary, restricted co-publication and co-citation between groups concentrating on viral pathogenesis (cluster #1) and those engaged in therapeutic research (clusters #0 and #5) may delay the translation of mechanistic insights into clinical applications. Future initiatives that intentionally finance or assemble translationally oriented teams, such as mandating collaboration between a virologist and an oncologist, could expedite the incorporation of fundamental research into clinical practice. The most frequently cocited author, Yuan Chang, has been referenced in 386 articles, with multiple studies citing more than one of their works. Among these, the most cited publication proposed that KSHV may act as a transforming virus in a unique subset of AIDS-related lymphomas and that KSHV and EBV may jointly influence B cells, promoting their malignant transformation (Cesarman et al., 1995). The two most cocited references are reviews that provide comprehensive summaries of KS research, laying the groundwork for further advancements in the field. However, these studies primarily rely on observational data and retrospective analyses, lacking the methodological rigor of randomized controlled trials. The third most cocited article offers a detailed analysis of KS risk among PLWH, enhancing the understanding of its associated risk factors. Cluster dependency analysis reveals that various research areas related to HIV-KS are closely interconnected, facilitating a more comprehensive understanding of the disease. Ongoing research should integrate research on #15 (viral genes) with other fields to promote interdisciplinary knowledge exchange. The overlay map indicates that, beyond molecular and biology, HIV-KS research intersects with multiple medical disciplines, displaying its vast influence within the medical field. The continuous presence of numerous keywords throughout co-occurrence, clustering, and citation burst analysis implies that these phrases have played a vital role in historical study and represent enduring themes warranting further analysis.

#### 4.1.2 Triangulation with previous bibliometric studies

To position our findings within a broader bibliometric framework, we compared the results of this HIV-KS analysis with other bibliometric studies on relevant topics. For example, Hao Zhang and colleagues conducted a bibliometric analysis on HIV and Alzheimer’s illness, indicating that the United States, Germany, and China regularly dominate in terms of publication production and centrality metrics (Zhang et al., 2025). We discovered that the United States’ prominent involvement in HIV-KS research closely matched its significance in the broader HIV research landscape. Similarly, several research hotspots identified in a study, such as “immune activation,” “antiretroviral therapy,” and “mortality” also emerged prominently in our HIV-KS network (Gong and Lai, 2023). These overlaps suggest that the structural features of collaboration in HIV-KS research are essentially consistent with those observed in general HIV-related bibliometric patterns.

In contrast, no previous study has focused completely on the bibliometric landscape of KS. Our work is the first attempt to systematically map the global research structure of KS using bibliometric methodologies. The developing trends and clusters highlighted in this study closely match the increasing research goals within the KS area. This congruence shows that our bibliometric findings not only give historical insights but also resonate with the forward-looking trend of research development in the field. Through triangulation with related literature, we confirm that HIV-KS research replicates broader macro-level patterns of research dominance among leading countries and regions, while also exhibiting a distinctive thematic breadth spanning from epidemiology to clinical treatment.

### 4.2 Research hotpots

Based on keyword clustering and citation bursts, current research hotspots can be categorized into four main areas. Epidemiological patterns and prevalence are evident in cluster #3 (seroprevalence), reflecting a sustained interest in epidemiological monitoring and burden estimation. Basic mechanism research includes cluster #1 (apoptosis), which examines fundamental biological and immunological processes. KSHV-related disease research contains cluster #2 (primary effusion lymphoma) and #6 (viral infections), focusing on the links between KSHV and HIV through diverse diseases. Treatments encompass cluster #0 (antiretroviral therapy), #4 (management), and #5 (pegylated liposomal doxorubicin), covering therapeutic and disease management approaches. Each category incorporates relevant citation burst keywords.

#### 4.2.1 Epidemiological patterns and prevalence

Serological testing is the primary method for assessing the prevalence of KS infection. It is widely used not only to identify high-risk groups and investigate the relationship between KSHV infection and KS but also to evaluate the impact of therapeutic interventions on the disease. KSHV is the causative agent of KS, and its seropositivity rate is closely correlated with the global prevalence of KS (Mesri et al., 2014). Epidemiological studies have demonstrated that KSHV prevalence varies significantly by geography, population group, and HIV status. The highest seroprevalence rates are found in sub-Saharan Africa, where KSHV seroprevalence is often already high in childhood, while prevalence is markedly lower in North America, Europe, and Asia (Jary et al., 2021). Among HIV-positive individuals, particularly men who have sex with men (MSM), KSHV seroprevalence is higher than in the general population, correlating with elevated KS incidence (Knights et al., 2023). These disparities reflect both differences in exposure routes and underlying immunosuppression, emphasizing the need for targeted surveillance in high-risk regions and populations. However, low KSHV antibody titers in individuals with low viral loads and cross-reactivity with other herpesviruses caused by antigenic similarities may lead to errors in serological testing (Mesri et al., 2010; Ryan and Rose, 2013), to accurately assess seropositivity, both latency-associated nuclear antigen (LANA) and at least one lytic antigen should be tested to capture variability in serological responses. Therefore, studying the pathogenesis of KSHV is essential for enhancing diagnosis, treatment, and overall understanding of the disease.

#### 4.2.2 Basic mechanism research

KSHV encodes various gene products with angiogenic and oncogenic properties, such as the viral FLICE-inhibitory protein (VFLIP), which inhibits Fas-mediated apoptosis, and the K1 protein. VFLIP can overexpress multiple cytokines and chemokines to drive the inflammatory response and induce defective B-cell differentiation and B-cell-derived tumors in genetically engineered mice. The HIV Nef protein is produced early during HIV infection and interacts with various cytokines to modulate cellular function. Both Nef and K1 activate the PI3K/AKT/mTOR signaling pathway, individually or synergistically, while reducing the level of Phosphatase and Tensin Homolog deleted on chromosome 10 (PTEN), a key tumor suppressor. They also induce miR-718 expression, which further inhibits PTEN, thereby promoting endothelial proliferation, angiogenesis, and tumorigenesis (Xue et al., 2014). HIV infection activates the nuclear factor kappa-light-chain-enhancer of activated B cells (NF-κB) signaling pathway, which inhibits KSHV reactivation in BCBL-1 cells. However, NF-κB pathway activation is generally linked to cell survival and resistance to apoptosis, and KSHV’s VFLIP can also activate this pathway, which is essential for maintaining the tumor phenotype (Ballon et al., 2011). Thus, it can be hypothesized that NF-κB activation may sustain cell survival by inhibiting apoptosis, thereby providing a stable cellular environment conducive to KSHV reactivation (Zhu et al., 2011). Correspondingly, the KSHV LANA protein interacts with the HIV transactivator of viral transcription protein to enhance transcriptional activity. In contrast, KSHV ORF45 and ORF50 proteins activate the HIV long terminal repeat sequence, thereby boosting HIV replication (Carbone et al., 2015). Collectively, these interplay reinforce viral persistence and tumorigenesis. However, many of these insights stem from *in vitro* or murine models, and it remains unclear how these interactions unfold *in vivo* within the immunosuppressed microenvironment. Future research should therefore prioritize translational studies that explore the therapeutic viability of simultaneously disrupting both viruses’ oncogenic signaling, potentially through inhibitors of common effectors like mTOR or NF-κB.

#### 4.2.3 KSHV-related disease research

KSHV-associated diseases (KADs) include KS, primary effusion lymphoma (PEL), KSHV-associated multicentric Castleman disease (MCD), and KSHV inflammatory cytokine syndrome (KICS). These diseases can co-occur and share similar characteristics, leading to multi-organ dysfunction (Cesarman et al., 2022; Hansen et al., 2022). PEL is a rare and aggressive B-cell non-Hodgkin lymphoma (Cesarman et al., 2022). It is often caused by co-infection with KSHV and EBV, which can infect and transform B cells (Liu et al., 2022). After KSHV infects B cells, it activates cell signaling pathways through the expression of viral proteins, such as vGPCR, vIL-6, and v-Cyclin, to promote B-cell proliferation and inhibit apoptosis (Andrei and Snoeck, 2015). EBV co-infection can also stimulate KSHV replication, thereby enhancing viral persistence and contributing to cellular transformation (Caduff et al., 2021; Böni et al., 2022). While both viruses contribute to transformation, KSHV exerts a stronger oncogenic influence, as its gHgL protein has a larger interaction surface and more binding sites with the ligand binding domain of human-related ligands (Lurain et al., 2024). Until now, there is no standard treatment protocol for PEL. Common treatments include the CHOP chemotherapy regimen (cyclophosphamide, doxorubicin, vincristine, and prednisone). And the study found that drugs in the CHOP regimen may interact with cART, exacerbating side effects (Flepisi et al., 2014). This underscores the therapeutic complexity of managing KADs, where viral oncogenesis and treatment-related interactions must be simultaneously addressed.

MCD is a lymphoproliferative disorder frequently associated with HIV infection. HIV infection compromises the host’s antiviral immune response, while KSHV capitalizes on the resulting immunosuppressive environment to evade immune surveillance, thereby establishing a self-perpetuating pathological cycle. Notably, vIL-6 encoded by KSHV activates downstream signaling pathways through direct binding to the glycoprotein130 (gp130) receptor subunit, effectively bypassing the classical receptor engagement and activating the JAK/STAT pathway to induce transducer and activator of transcription 3 (STAT3) phosphorylation and acetylation, which further promotes cellular proliferation, angiogenesis, and systemic inflammatory responses (Shimoda et al., 2023). These severe situations remain the leading cause of death among HIV-positive MCD patients (Broccolo et al., 2016). KICS is an inflammatory disorder caused by KSHV, with MCD-like symptoms but without its histological characteristics, and is considered by some as a potential precursor to MCD (Carbone et al., 2015). These discoveries illustrate how KSHV exploits immune dysfunction to drive a range of overlapping inflammatory and neoplastic syndromes; acknowledging this shared etiology supports a theoretical change from treating these conditions in isolation to managing them as a common viral disease complex.

#### 4.2.4 Treatments for Kaposi’s sarcoma

The treatment of KS requires a comprehensive approach, particularly in the context of HIV infection. The combined use of ART, chemotherapy, and effective patient management is essential. In KS patients, ART enhances KSHV-specific CD8 T-cell responses, which suppress KSHV replication and reduce the incidence of KS (Bourboulia et al., 2004). Despite ART’s effectiveness in controlling HIV, it does not fully restore immune function, and chronic inflammation persists. Consequently, PLWH remain at higher risk for cancer, particularly cancers related to immunosuppression (Hernández-Ramírez et al., 2017). Additionally, the adverse effects of ART and the emergence of HIV drug-resistant mutations present ongoing challenges in treatment. These limitations illustrate that immune reconstitution does not equate to immune normalization and the continued burden of KS.

In addition to ART, chemotherapeutic agents such as pegylated liposomal doxorubicin (PLD) and paclitaxel are commonly used in the treatment of established KS. PLD is considered a first-line treatment, especially for advanced or aggressive forms of KS. However, due to its cardiotoxicity, careful monitoring of the patient’s cardiac function is required during treatment (Dzobo, 2021). Other chemotherapeutic agents, such as paclitaxel, have also shown effectiveness in treating KS. Randomized controlled trials indicate that paclitaxel monotherapy has a slightly higher response rate and more prolonged progression-free survival compared to PLD, although it is associated with significantly increased toxicity (Robey and Bower, 2015). Patient management is critical to KS treatment, particularly for patients with severely compromised immune function. Immune Reconstitution Inflammatory Syndrome (IRIS) is an inflammatory response in PLWH receiving ART, triggered by immune system recovery. Research by Rebecca C. and colleagues indicates that in some African regions, the mortality rate for KS-IRIS can be as high as 48% (Robey and Bower, 2015). Current management of IRIS relies primarily on nonsteroidal anti-inflammatory drugs. Emerging strategies, such as initiating ART earlier and providing preemptive prophylaxis in high-risk patients, highlight a shift toward preventive and risk-adapted models (Nelson et al., 2017). These findings point to the broader therapeutic complexity of HIV-KS: clinicians must manage two interacting viral diseases, mitigate drug-related toxicities, and address inflammatory complications. This underscores the urgent need for integrated treatment frameworks that are both biologically targeted and resource sensitive, especially in regions where KS prevalence remains high but access to chemotherapy and side effects management is limited.

### 4.3 Frontiers

Based on the potential upward trends observed in the landscape map and the citation bursts that have persisted until recently, emerging trends and research frontiers in current HIV-KS studies include PLD, paclitaxel, diagnosis, prevalence, and related topics.

#### 4.3.1 Emerging trends in Kaposi’s sarcoma prevalence and diagnosis

Current research prioritizes understanding evolving prevalence patterns, particularly by examining variations in disease rates across different populations, with a focus on epidemiological studies among MSM. A study by Rebecca Monica Tibenderana in collaboration with others indicated that in sub-Saharan Africa, men have a higher infection risk than women. However, no significant gender differences were observed in child populations or regions such as Asia, Europe, and North America (Tibenderana et al., 2024). Among MSM, KSHV infection rates are higher and strongly associated with specific sexual behaviors and HIV status (Knights et al., 2023; Li et al., 2023; Salyards et al., 2024). In frontier research on KS diagnosis, Jason Cade Manning and colleagues recently proposed a simplified, POC-compatible alkaline DNA extraction method, ColdSHOT (Manning et al., 2024). Compared to previous methods, it offers a rapid, cost-effective, and user-friendly DNA extraction technique. In the domain of prognosis, Qian and colleagues have devised a nomogram utilizing a competing risk model to assess the risk of KS-specific death in patients with cutaneous KS This model covers critical parameters such as age, year of diagnosis, and race, and is created employing the Fine–Gray competing risk approach. It has shown great accuracy and reliability in validation (Qian et al., 2023). The nomogram helps provide personalized risk assessment, guides clinicians in choosing suitable therapies, and serves as a useful reference for developing comparable tools for wider patient populations.

#### 4.3.2 Advancements in therapeutic strategies for Kaposi’s sarcoma

Research on KADs has also identified potential therapeutic targets for KSHV. Inhibition of serine–threonine liver kinase B1 (LKB1) induces apoptosis in infected cells (Li et al., 2024). These findings offer new therapeutic implications for KSHV-associated diseases, and recent PLD research emphasizes its synergistic effects with other drugs in KS treatment. Catherine B Goff and collaborators have found that combining PLD with single-agent bevacizumab and other drugs enhances its therapeutic efficacy (Goff and Dasanu, 2023). Research on paclitaxel has focused on improving its effectiveness and minimizing side effects through nanotechnology (Mustafa et al., 2023). A case report by Lele Yu et al. highlighted the efficacy and safety of Nab-paclitaxel in treating recurrent AIDS-KS. It also reported that serum concentrations of Nab-paclitaxel were 10 times higher than those of regular paclitaxel, while intratumoral concentrations were 33% greater at the same dose (Yu et al., 2024). However, due to the limitations of case reports, the efficacy of Nab-paclitaxel still needs to be further verified by large-scale, multi-center clinical studies. New therapies, such as electrochemotherapy (ECT), have also demonstrated significant efficacy in treating KS, which was reflected in its well tolerated, with a low complication rate (Rullo et al., 2024). Additionally, long-pulsed neodymium-doped yttrium-aluminum-garnet (Nd:YAG) laser therapy has shown notable clinical and dermoscopic improvements (Bostancı et al., 2024). On May 15, 2020, the U.S. Food and Drug Administration expanded the approval of pomalidomide for the treatment of KS, particularly in patients who have developed resistance to ART. However, research on it remains limited, and further trials are necessary to assess its therapeutic potential and possible side effects.

### 4.4 Obstructions and prospects

#### 4.4.1 Challenges in global research equity

In HIV-KS research, institutional partnership networks exhibit a strong core-periphery difference. High-resource institutions like the USA-NIH and the University of California dominate funding and authorship, while research facilities in high-burden countries such as sub-Saharan Africa frequently function largely as sample providers, with limited responsibilities in study design, analysis, and publishing. This disparity suppresses locally relevant research on low-cost solutions and reinforces one-way knowledge flows, where central institutions’ reviews are often acknowledged, but local studies gain limited acknowledgment. Underlying factors include chronic underfunding, inadequate infrastructure, and insufficient diagnostic and laboratory capability. In addition, bureaucratic ethics approvals, language obstacles, and publication restrictions severely impede the academic contributions of academics in these countries. Future efforts should prioritize equitable research collaborations by expanding research and educational opportunities in resource-limited regions. Equally important is a shift from an “aid-based” model to one of mutual benefit, recognizing the value that endemic regions bring through rich epidemiological data and large patient populations. These contributions can enhance global models and guide relevant clinical studies. Such reciprocal value exchange strengthens the sustainability and impact of global collaboration. Journals should also require the inclusion of authors from endemic regions in multicenter studies. To overcome current gaps, it is necessary to construct regional innovation centers that integrate molecular, clinical, and epidemiological research, and to develop integrated databases incorporating viral genomes, clinical phenotypes, and epidemiological data. Equitable participation can be advanced by coordinated efforts in finance, data exchange, and capacity building, enabling local evidence to drive practice. Adopting new technologies is also crucial. Approaches like target enrichment and transcriptomic workflow-TRIMD can help profile the KSHV transcriptome in African patients, potentially finding novel transcript variations and therapeutic options for endemic KS (Shekhar et al., 2024). These efforts will foster a more inclusive and sustainable future for HIV–KS research globally.

#### 4.4.2 Priorities for pathogenesis and treatment

While experimental research has elucidated some mechanisms of KS pathogenesis, these studies have limitations, including small sample sizes and reliance on specific models that may not fully reflect the complexities of *in vivo* environments. Future research should combine multiple experimental models and validate findings using human samples, such as B cells from KSHV-infected patients. The following research should focus on optimizing drug dosages and personalized treatment strategies to minimize side effects and exploring the combined use of drugs with PD-1 inhibitors and targeted therapies. Considering the continued prominence of keywords such as apoptosis and antiretroviral therapy, future research may benefit from investigating how ART regulates apoptotic pathways in KSHV-infected cells, an underexplored mechanism that may play a role in KS regression. Special attention should be given to specific patient populations, including the impact on female fertility and pediatric growth and development. Shifts in research themes frequently align with significant policy modifications or financial redistributions. Future bibliometric analyses may use funding data to enhance the prediction of future hotspots, an approach especially beneficial for forecasting planned advancements, such as anticipated investments in KSHV vaccine research. Additionally, we observe a potential “short-term problem-solving bias” in the field: when KS incidence rises, research efforts tend to focus on epidemiological surveys and clinical management guidelines to satisfy immediate requirements, while investment in core pathogenesis studies declines. Over time, this produces a vicious cycle because without fresh mechanistic discoveries, pharmaceutical development stagnates, or research reaches a bottleneck with no significant progress. As a result, attention shifts back to the disease’s heavy burden and epidemiology, once again drawing focus toward short-term clinical responses. Breaking this cycle requires targeted funding mechanisms to sustain fundamental research clusters and support the discovery of breakthrough advances. Future funding should also target critical knowledge gaps, including the discovery of early diagnostic markers, mechanisms of drug resistance, and the development of targeted therapies (Valantin et al., 2022). Additionally, collaborative projects that connect basic research with clinical applications should be actively supported. Finally, with support from the NIH – USA, efforts should be intensified to develop a KSHV vaccine.

## 5 Limitations

This study has several limitations. First, the articles were obtained exclusively from the WOSCC, and the analyzed data lacked comprehensiveness, as other databases were not included. Second, as the research field evolves, future advancements in HIV-KS studies may lead to discrepancies between bibliometric analyses and actual research trends. Thirdly, the analysis may overlook recent studies or underrepresent less cited yet potentially significant works. In addition, the study design and the software tools used limit our ability to fully explore statistical interactions, confounding factors, or hierarchical associations between or across the variables. While we have attempted to mitigate this limitation through manual verification, the potential for misinterpretation analysis remains. This limitation may influence the interpretation of some visual findings.

## 6 Conclusion

Despite the widespread use of ART, the high incidence of KS and its impact on health remain a significant concern. This study provides a comprehensive bibliometric analysis of HIV–KS research, outlining the intellectual structure, knowledge evolution, and collaboration networks of the field. The study reveals the current state of research in the field and identifies key hotspots. In addition, the results reveal persistent inequalities in institutional influence and regional participation, particularly the underrepresentation of scholars and institutions from high-burden areas. Addressing these disparities requires targeted strategies. Future research should focus on matching molecular studies with clinical demands, fostering interdisciplinary approaches, optimizing integrated treatment options for HIV-KS, and stimulating innovation in diagnostics and medicines. Policymakers and funding agencies could support this by prioritizing low and middle-income countries-led initiatives and investing in context-specific research infrastructures. These efforts will help realign research priorities with global disease burdens and advance more equitable, evidence-based strategies for the prevention and treatment of HIV-KS.

## Data Availability

Publicly available datasets were analyzed in this study. This data can be found at: https://webofscience.clarivate.cn/wos/woscc/summary/7ad31a26-bb15-4896-86e7-e13526f1a767-0156f789e4/relevance/1.
